# Student nurses experiences of moral distress: A concept analysis

**DOI:** 10.1111/jan.16370

**Published:** 2024-08-05

**Authors:** Timmins Rebecca

**Affiliations:** ^1^ University of Wolverhampton Wolverhampton UK

**Keywords:** clinical practice, concept analysis, ethics, internal constraints, moral courage, moral distress, nurse education, nurses, student

## Abstract

**Aim:**

To understand how pre‐registration student nurses experience moral distress and refine the concept in this population.

**Background:**

The experience of moral distress has positive and negative effects for health professionals and negatively impacts on patient care. Moral distress is a fluid concept which permits the experience to be varied among different populations. Despite empirical research, a concept analysis has not been performed in the student nurse population.

**Data Sources:**

Electronic databases were searched via Ebsco Host Complete and included Cinahl, Medline, APA Psych in March 2024. Search terms included ‘Moral Distress’ AND ‘Student’, ‘Moral Distress’ and ‘Baccalaureate.’ Search limits included articles between 2014 and 2024, English Language. Twenty‐five papers were included in the review and consisted of eight quantitative studies, 11 qualitative studies, three mixed methods studies and three literature/systematic reviews.

**Methods:**

An integrated mixed research synthesis (Sandelowski, Voils, Barroso 2006) was conducted and organized into Walker, Avant's (2005) framework of antecedents, attributes and consequences. Braun and Clarkes (2006) thematic analysis was then used to generate themes from the literature.

**Results:**

Antecedents emerged as students having moral sensitivity, they recognize unethical circumstances. Attributes identified roots of moral distress. These roots include poor patient care, harm to the patient and unsafe care. Students experience of morally reprehensible events is exacerbated by the disempowerment they experience as being ‘just a student’. Student nurses who do not exhibit moral courage and do not oppose immoral practices do so due to internal constraints which transpire as fear of conflict, withdrawal of learning opportunities, and fear of disruption to learning. This is influenced by their registered nurse supervisor relationship. Consequences of moral distress identify negative feelings, coping mechanisms and positive effects.

**Conclusion:**

The attributes of moral distress in the student nurse population have distinctive features which should be considered by nurse educators and in empirical research.

**Patient or Public Contribution:**

None, as this is a concept analysis that contributes to theory development and is not empirical research.

## 1 INTRODUCTION

Ethical problems can arise for nurses internationally in at least two different ways: moral dilemmas and moral distress (Jameton, 1984; Wilkinson, 1988). Moral distress was first introduced into the nursing literature in 1984 where it was identified by Andrew Jameton that moral distress occurs when one knows the right thing to do, but institutional constraints or, conflicts with co‐workers make it impossible to pursue the right course of action (Jameton, 1984).

Numerous accounts of the negative effects of moral distress have been reported internationally. Health care professionals have reported feelings of guilt and anguish (Gandossi et al., 2023), a lost capacity for caring, avoidance of patients, provision of poor patient care, and development of physical and psychological problems as a result of this phenomenon (Rushton, 2006; Wilkinson, 1988). Student nurses have experienced frustration and disappointment which has emanated from the powerlessness felt by the students when they find themselves facing unethical behaviours of other healthcare professionals (Escolar Chua, Magpantay 2019). Increased patient pain, longer hospital stays, and inadequate care are reported in the patient population (Corley, 2002), due to communication skills, lack of trust, defensiveness, and increased interprofessional conflict in the nursing profession (Rushton, 2006).

## 2 BACKGROUND

In nursing, the empirical evidence has predominantly focused upon moral distress in critical care nursing (McAndrew & Harding, 2020) but has also explored the phenomenon in other disciplines including nursing professors (Toescher et al., 2020), correctional nurses (Lazzari et al., 2020) and student nurses (Bordignon et al., 2019). Different professionals, however, have dissimilar moral agency, and the capacity to engage with moral knowledge, skills, and actions depending upon their role (Lutzen & Kvist, 2012). It is suggested that nurses can have different experiences of moral distress than physicians due to the decision‐making hierarchies in healthcare leaving nurses more likely to be constrained by the decision making of others (Fourie, 2016). Within the student nurse population empirical evidence suggests students are viewed as being at the very bottom of hierarchies and this contributes to their moral distress and increased risk of depression and suicide risk (Paidipati et al., 2023).

The lack of clarity regarding the different attributes of moral distress in different contexts reflects the fluidity of the concept (Russell, 2012). Agreement exists that moral distress is a fluid and context dependent experience (Hardingham, 2004; Wilkinson, 1988). Within healthcare, concept analysis of moral distress has been performed within the specialities of neuroscience nursing (Russell, 2012), critical care nursing (Cooke et al., 2022) and Midwifery (Foster, 2022). Despite considerable empirical research on moral distress within the population of student nurses, there has not yet been a concept analysis performed in this population. Theory used to develop the empirical evidence base in the student nurse population has often derived from the registered nurse population. This concept analysis therefore seeks to explore how student nurses experience moral distress and how this is different to other populations.

## 3 METHODOLOGY

The concept analysis is based upon Walker and Avant (2005) method which derives from Wilson's (1968) work which adopts a constructivist approach (Duncan et al., 2007). Philosophically, a constructivist approach is consistent with understanding student nurses' experiences of moral distress.

Data was extracted, analysed and added to the Walker and Avant (2005) 8 step framework using a integrated mixed methods synthesis (Sandelowski et al., 2006). Integrated designs enable papers to be grouped for synthesis not by methods (i.e. qualitative and quantitative), but rather by findings viewed as answering the same research questions or addressing the same aspects of a target phenomenon (Sandelowski et al., 2006). Data from the mixed methods synthesis was then themed and analysed using Braun and Clarke (2006) Thematic Analysis.

The aim of the concept analysis was to understand how student nurses experience moral distress and refine the concept in this population.

## 4 DATA SOURCES

**FIGURE 1:**
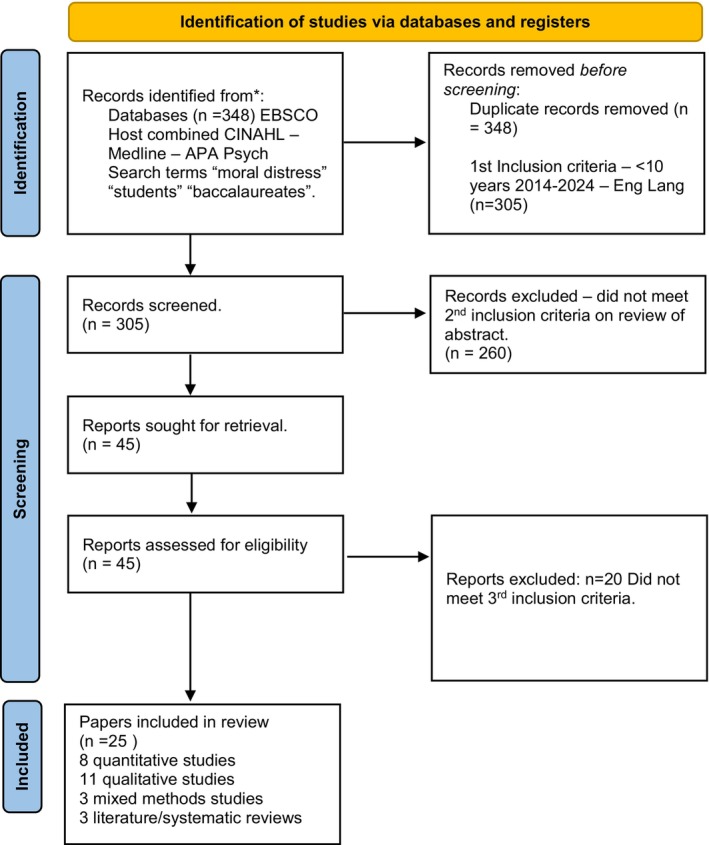
PRISMA flow diagram summarising the screening process. First inclusion criteria—Eng Lang and <10 years. Second inclusion criteria—peer reviewed literature reviews, primary research papers and systematic reviews identifying either attributes, antecedents and consequences of moral distress in pre‐registration student nurses in abstract. Third inclusion criteria—on full text review of the peer reviewed paper (lit review, primary research, systematic review) attributes, antecedents and consequences of moral distress in pre‐registration student nurses were identified and included.

In March 2024 the researcher consulted Electronic databases and searched via Ebsco Host Complete and included Cinahl, Medline, APA Psych. Search terms included ‘Moral Distress’ AND ‘Student’, ‘Moral Distress’ AND ‘Baccalaureate.’ 1st inclusion criteria applied were articles published between 2014 and 2024, English Language. Three hundred and five papers were yielded and reviewed against inclusion criteria (See Figure 1). 2nd inclusion criteria required peer reviewed primary research papers, systematic or literature reviews that identified either attributes, antecedents, or consequences of moral distress in the student nurse population. Forty‐five papers were initially shortlisted against the 1st and 2nd inclusion criteria by reviewing the abstract. On full text review of the 45 peer reviewed papers 25 papers were included in the concept analysis as they met 3rd inclusion criteria, being attributes, antecedents and consequences of moral distress in pre‐registration student nurses were identified in the paper and included in the analysis. This consisted of eight quantitative studies (See Table 1), 11 qualitative studies (See Table 2), three mixed methods studies (See Table 2) and three literature/systematic reviews (See Table 2).

**TABLE 1:** Quantitative studies included in the concept analysis.

Author	Goals	Country	Design and sample	Data collection	Results
Bordignon, Lunardi, Barlem et al. (2019)	To analyse moral distress and its relation with sociodemographic and academic variables in undergraduate students	Brazil	Cross‐sectional design.‐499 nursing students from three universities in Brazil. (Students year 1–3)	Survey—Moral Distress scale for Nursing Students – Survey	The demographic and academic characteristics of the undergraduate nursing students who referred higher levels of moral distress were being enrolled in the final course semesters, were at a federal university, and had no prior degree as an auxiliary nurse/nursing technician.
Escolar Chua (2018)	To explore the relationship between and among moral distress, moral sensitivity, and moral courage of undergraduate baccalaureate nursing students.	Philippines	Descriptive‐correlational design—293 baccalaureate Filipino nursing students (does not state student year of study).	3 Surveys MDR‐R Revised Moral Sensitivity Questionnaire Professional Moral Courage (PMC) Scale	Majority of students in the clinical areas encounter morally distressing situations that compromise quality patient care. Students perception of being the inexperienced among the healthcare team drives the majority of them to ignore morally distressing situations to avoid conflict and confrontation.
Gibson, Duke, Alfred (2020)	Determine the impact of moral resilience and moral courage on moral distress.	United States	Descriptive correlational study – 45 students/ 36% response rate (does not student year of study).	3 Surveys Moral Distress Thermometer (MDT), the Connor‐Davidson Moral Resilience Scale (CD‐RISC), and the Moral Courage Scale for Physicians (MCSP).	Students reported mild levels of moral distress. Moral resilience was significantly correlated with moral courage, age, and students having a previous degree.
Janatolmakan, Dabiry, Rezaeian et al. (2021)	Causes, frequency and intensity of MD in student nurses. What is the relationship with Moral Distress theory (MDT)	Iran	Cross‐sectional study, 68 student nurses (does not state year of study).	Moral Distress Scale‐revised (MDS‐r)	A statistically significant relationship between the gender, work experience, and participation in the professional ethics workshop variables and mean MDS‐total and MDT was evident.
Krautsheid, DeMeester, Orton et al. (2017)	To assess moral distress among senior baccalaureate nursing students, describe ethical dilemmas contributing to their moral distress in practice settings, and identify reasons for inaction when encountering dilemma.	US	Descriptive cross‐sectional survey design. 267 student nurses.(Senior level nursing students)	MDT Demographics Content Analysis	Aggregate mean moral distress rating was 3.12. Content analysis revealed compromised best practices, disrespect for human dignity, perceived constraints, and navigating personal values. The most frequent reasons for inaction were subordinate role, relationship preservation, incomplete knowledge, and uncertainty about speaking up.
Krautsheid, Mood, McLennon et al. (2020)	To examine relationships between resilience protective factors and moral distress associated with clinical practice in nursing students.	US	A correlation pilot study. 60 student nurses. (Senior level nursing students)	Scale of Protective Factors and MDT Survey.	Aggregate moral distress rating was x = 3.67. Two of four resilience protective factor subcategories demonstrated significant inverse correlations with moral distress rating. Inverse correlations were found between social support and moral distress, and between goal efficacy and moral distress.
Mazzotta, DeMaria, Bove et al. (2022)	To translate, culturally adapt and evaluate the psychometric properties of an Italian version of the Moral Distress Scale for Nursing Students (It‐ESMEE) for use with delayed nursing students (students who could not graduate on time or failed the exams necessary to progress to the next level).	Italy	Cross‐sectional research design. 282 delayed nursing students. (Delayed nursing students).	Moral Distress Scale for Nursing Students (It‐ESMEE).	The study confirmed a multidimensional second‐order factorial structure for the It‐ESMEE with five dimensions: improper institutional conditions to teach user care, authoritarian teaching practices, disrespect for the ethical dimension of vocational training, lack of competence of the teacher and commitment of ethical dimension of user care.
Paidipati, Lozano, West et al. (2023)	To understand the mediating effect of depression on the relationship between moral distress and suicide risk among undergraduate nursing students.	US	Cross‐sectional analysis was derived from a larger sequential mixed methods study. 679 Nursing Students. (Both junior and senior nursing students).	Moral Distress Thermometer (MDT), Depression Scale‐Revised (CESD‐R), Suicidal Affect‐Behaviour‐Cognition Scale (SABCS), and the Brief Social Desirability Scale (BSDS). Surveys.	The relationship between moral distress and suicide risk was fully mediated by depression and statistically significant at the alpha = 0.05 level. All three psychological variables (depression, moral distress, suicide risk) impact nursing students and require innovative solutions within nursing and educational programs.

**TABLE 2:** Qualitative studies, Literature reviews/systematic reviews and mixed methods studies included in concept analysis.

Author	Goals	Country	Design and sample	Data collection	Results
Qualitative studies					
Bahramirad, Heshmatifar, Rad (2020)	Aimed to discover and describe the problems and benefits of the night shift for nursing internship students.	Iran	Qualitative content analysis design. 15 semester 7/8 students.	Conventional content–analysis.	Seven main categories were extracted, five of which included problems such as (1) exploitations (2) being an outsider, (3) moral distress (4) learning deficit and (5) annoyance.
Bongiorno et al. (2023).	Researchers sought to examine nursing students' and new graduate nurses' impressions of the nursing profession in contrasting regions of New York State during the first wave of the COVID‐19 pandemic.	US	Inductive content analysis was performed on narrative text responses drawn from a larger multisite mixed‐methods survey. 295 newly graduated/student nurses (students n‐197).	Inductive content analysis.	Five sub concepts were abstracted, leading to the main concept of shocked moral distress. Nursing students and new graduate nurses have experienced high levels of moral distress but remain committed to the profession.
Bordignon, Lunardi, Barlem et al. (2018)	To understand the resistance strategies adopted by undergraduate students in nursing, faced with situations of MD.	Brazil	Qualitative, exploratory, descriptive research. 21 undergraduate student nurses, Brazil, 3 different Universities. (Students from years 1,2,3)	Discursive textual analysis and Foucauldian theoretical reference.	Students resisting demonstrate a sense of self‐preservation and moral empowerment. Non‐resistance initiatives are related to the fear of possible sanctions. Thus, by resisting or not, students may experience both positive and negative repercussions.
Escolar Chua, Magpantay (2019)	To explore the moral distress experiences encountered by undergraduate baccalaureate nursing students in community health nursing.	Philippines	A descriptive qualitative design. 14 Senior Nursing Students. (Senior nursing students)	In depth Interviews.	Findings of the study surfaced three central themes which included (1) moral distress emanating from the unprofessional behaviour of some healthcare workers (2) resulting sense of powerlessness (3) differing values and mindsets of the people they serve in the community.
Gandossi et al. (2023)	Aims to analyse episodes of moral distress experienced by nursing students during end‐of‐life care of onco‐ hematologic patients in hospital settings.	Italy	Hermeneutic phenomenology. 17 second‐ and third‐year Nursing students. (3rd year students)	Qualitative interview. Interpretative Phenomenological Analysis.	Eight themes identified causes of moral distress; (1) factors that worsen or influence the experience of moral distress (2) feelings and emotions in morally distressing events (3) morally distressing events and consultation (4) strategies to cope with moral distress (5) recovering from morally distressing events (6) end‐of‐life accompaniment (7) internship clinical training, and (8) nursing curriculum.
Kane, Wareing, Rintakorpe (2022)	The psychological impact of deployment on final‐year nursing and midwifery students working in the NHS during an international public health crisis.	UK	53 third‐year student adult, paediatric and mental health nurses and midwives were surveyed out of a cohort of 246. (3rd year students).	Survey content analysis—Maslach burnout inventory—Free text questions evaluated and edited. Constant analysis method to generate themes.	Overall, students found the experience of being deployed into clinical practice during a major public health emergency a valuable and unique experience that strengthened their resilience. However, students reported a significant level of personal obligation to opt‐in to deployment. Working within clinical areas caused heightened anxiety and uncertainty, which was alleviated by managerial support.
Mæland, Tingvatn, Rykkje et al. (2021)	The aim of this article is to provide knowledge of moral distress in clinical nursing practice.	Norway	Bachelor students (n‐19) and further education (n‐17) nursing students were invited to write a story about challenging situations from their own clinical practice, resulting in 36 stories. (Does not specify student year of study).	Hermeneutical reading by researchers of stories. Researchers selected six narratives to illuminate the phenomenon of moral distress in clinical practice	Bachelor students and further education students shared similar stories dealing with moral distress in clinical practice.
Reader (2015)	This qualitative study aimed to describe experiences of moral distress among students enrolled in associate degree nursing program.	US	15 nursing students purposive sampling.	Interviews. Thematic: across‐case analysis and narrative: within‐case analysis.	Themes that emerged were (1) identification of stress and moral distress. (2) learning and working in an unjust culture. (3) disempowerment and status, (4) moral reside and regret.
Renno, Ramos, Brito (2018)	To identify the existence of moral distress caused by ethical conflict and dilemmas experienced during their nursing education.	Brazil	Qualitative multiple‐case study. 58 student nurses. (Does not state student year of study).	FGD's and thematic analysis.	Themes that emerged: (1) moral distress is experienced by undergraduates in the reality of healthcare services, (2) the teacher as a source of moral distress, and (3) moral distress as a positive experience.
Wojtowicz, Hagen, Van Daalen‐Smith (2014)	Investigate the experience of moral distress in inpatient psychiatric settings, from the nursing educational perspective.	Canada	Naturalistic Qualitative design. 8 Mental Health Nursing Instructors who assisted students who experienced MD.	2 hour long FGD with 4 participants in each FG. Inductive Thematic Analysis performed. (2nd/3rd year students).	Three major themes emerged: (1) ‘taking a stand.’ (2) ‘they don't know the whole picture’, and (3) ‘I'm a guest in the house’ Instructors witnessed many of the same moral issues that the students identified, such as an over‐emphasis on psychiatric medications (as opposed to talking to patients), the hierarchical status of psychiatrists on the units, and deceiving and/or withholding information from patients.
Yilmaz and Kiziltepe (2022).	To explore the moral distress experiences encountered by undergraduate nursing student.	Turkey	Phenomenology. 126 Senior Nursing Students (3rd year).	Data collection form was prepared to determine the descriptive characteristics of the students and 4 open‐ended structured questions.	Three themes were identified: (I) Situations that cause moral distress in students (Medical error, neglect of duty, negative behaviour to patients and colleagues) (II) Student feelings and emotions (distrust, resentment, anger) (III) Student attitudes and behaviours (question their profession, silence).
Literature and systematic reviews					
Bickhoff, Sinclair, Levett‐ Jones (2017)	To explore factors which facilitate or inhibit undergraduate nursing students' willingness to demonstrate moral courage when confronted by poor patient care.	N/A	15 Qualitative research papers that explored undergraduate nursing students' depictions of situations where moral courage was or was not demonstrated during clinical placements. (1st, 2nd and 3rd year students)	Literature review. CASP analysis.	Despite feeling a moral obligation to act, most nursing students lack the moral courage to intervene or speak up when faced with poor practice. While students may subsequently report the behaviour, at the time of the event, they often remain passive spectators and sometimes even active participants. The major themes identified in the literature were: just a student, don't rock the boat, fear of consequences, mentor—student relationship, and patient advocate identity. The literature also identified that nursing students suffer ongoing moral distress when they do not have the courage to confront poor practice.
Heng T, Horey S (2023)	Experiences of Moral Distress in nursing students – A qualitative Systematic Review	N/A	7 Qualitative Studies were included.	Systematic review.	Four main themes: (1) Inadequacy and lack of autonomy, (2) Unprofessionalism of healthcare professionals, (3) Differing cultural views and values of patients and their relatives, and 4) Healthcare needs versus resource constraints.
Sasso, Bagnasco, Bianchi et al. (2016)	To describe how dilemmas and environmental, relational, and organizational factors contribute to moral distress in undergraduate student nurses.	N/A	Four articles matched the search criteria (one quantitative study and three qualitative) and included in the review. (Does not state year of student study)	Systematic Review	Inequalities and healthcare disparities, the relationship with the mentor, and students' individual characteristics can all impact negatively on the decisions taken and the nursing care provided, generating moral distress.
Mixed Methods studies					
Defilippis, Prati, Scascighini (2023)	To investigate the moral distress and the ethical issues most often encountered by nursing students during the first wave of the COVID‐19 pandemic in southern Switzerland.	Switzerland	Mixed Methods (n‐102 student nurses). (Does not state student year of study).	Convergent design. MDS‐R Survey and thematic analysis.	Main source for moral distress was ‘poor teamwork’ and issues related to appropriateness of care and working conditions, with a clear reference to students' own safety and that of their loved ones; the other concerns reported included the loss of learning opportunities and the perceived lack of technical knowledge and skills.
Feeg, Mancino, Rushton et al. (2021)	Ethical Dilemmas for Nursing Students and Faculty: In Their Own Voices	US	Students (*n* = 428) and faculty (*n* = 476) (senior nursing students).	Survey – Quant and Qual questions.	Data exemplify the differences in perspectives from students and faculty in their own voices around their perceptions of ethical dilemmas and their perceptions of ethical content in the curriculum. The voices of students suggest that the cognitive dissonance in what they believe is ethically wrong, perpetrated by those they might respect or look up to, produces the visceral moral discomfort heard in their lengthy descriptions of clinical situations.
Mehdipour Rabouri, Dehgan, Nematollahi (2018)	Nursing students' ethical challenges in the clinical settings: A mixed methods study.	Iran	To explore and to evaluate the nursing students' ethical challenges in the clinical settings in Iran	Exploratory sequential design. Qualitative (n‐37 students) first to develop tool for survey (n‐ 20 students).	Three main categories were extracted from qualitative data including Low attention of nurses to the patients' preferences; Lack of authority; and Inadequate support.

Braun and Clarke (2006) thematic analysis was used to manually code the data in the literature (See Table 3). This procedure has the advantage of not incorporating the researcher's analytic preconceptions within a deductive pre‐existing coding frame and is a flexible approach that can be used across a range of epistemologies.

**TABLE 3:** Themes emerged using Braun and Clarke (2006) Thematic analysis organized into Antecedents, Attributes and Consequences.

Antecedents of moral distress	Themes	Sources
Moral Sensitivity	Identification of moral conflicts and implications of these. Rejection of morally reprehensible situations. Ethics education influencing moral sensitivity.	Bordignon, Lunardi, Barlem et al. (2018) Escolar Chua, Magpantay (2019) Janatolmakan, Dabiry, Rezaeian et al. (2021)
Attributes		
Roots of moral distress: Poor patient care.	Witnessing poor care. Conforming in poor care. Poor staffing and lack of continuity of care impacting on poor care. Over or under medicating unnecessarily. Poor communication influencing poor care. Supervisor—student relationship influencing quality of care. Student disempowerment. Patients treated with lack of dignity.	Bahramirad, Heshmatifar, Rad (2020) Bickhoff, Sinclair, Levett‐ Jones (2017) Defilippis, Prati, Scascighini (2023) Escolar‐Chua (2018) Escolar Chua, Magpantay (2019) Gandossi et al. (2023) Janatolmakan, Dabiry, Rezaeian et al. (2021) Mæland, Tingvatn, Rykkje et al. (2021) Mehdipour Rabouri, Dehgan, Nematollahi (2018) Renno, Ramos, Brito (2018) Wojtowicz, Hagen, Van Daalen‐Smith (2014) Yilmaz and Kiziltepe (2022).
Roots of moral distress: Harm to the patient.	The Dr's wrongdoing causing harm. The disease harming the patient. Treatment not given in patient's best interests and influenced by medical staff and family interests. Hierarchical structures difficult to challenge.	Escolar – Chua (2018). Gandossi et al. (2023). Mæland, Tingvatn, Rykkje et al. (2021) Renno, Ramos, Brito (2018)
Roots of moral distress: Witnessing and/or participating in unsafe care.	Witnessing incompetence in care delivery. Feeling incompetent to carry out care. Practicing without adequate PPE, feeling dispensable. Poor working conditions giving rise to poor care. Unsafe staffing levels influencing poor care. Student disempowerment. Nursing practice being carried out differently, not to standard taught in university.	Bahramirad, Heshmatifar, Rad (2020) Bickhoff, Sinclair, Levett‐ Jones (2017) Bongiorno et al. (2023). Defilippis, Prati, Scascighini (2023) Escolar – Chua (2018) Janatolmakan, Dabiry, Rezaeian et al. (2021) Kane, Wareing, Rintakorpe (2022) Mæland, Tingvatn, Rykkje et al. (2021) Renno, Ramos, Brito (2018) Yilmaz and Kiziltepe (2022).
Roots of moral distress: Immoral activity in the academic environment.	Authoritarian teaching practices. Incivility. Teachers ethical attitude. Students feeing threatened, punished, humiliate by academic staff. Teachers research preference over teaching. Students own behaviours impacting each other. Cheating, copying, bullying. Disempowerment.	Bordignon, Lunardi, Barlem et al (2018). Janatolmakan, Dabiry, Rezaeian et al (2021). Mazzotta, DeMaria, Bove et al (2022). Reader (2015). Renno, Ramos, Brito (2018). Sasso, Bagnasco, Bianchi et al (2016).
Roots of moral distress: Inequalities in health.	Witnessing poor living conditions. Witnessing lack of access to healthcare. Lack of basic social necessities. Lack of safe and clean drinking water. Children being exposed to unsafe environments. Child neglect. Families working day and night to feed their family. Parents putting their own health last. Adolescent pregnancies.	Escolar, Magpanatay (2019). Sasso, Bagnasco, Bianchi et al (2016). Yilmaz and Kiziltepe (2022).
Roots of moral distress: Disparity between taught ethical principles and unethical practices in clinical practice.	Bioethical principles taught in university were violated in practice. Confidentiality breached. Injustices witnessed. Patients treated differently based upon their financial position. Health care staff rude to patients. Demoralizing and dehumanizing treatment of patients. Concerns of psychological trauma of patients. Patients' negative encounters with Health staff may discourage seeking further medical help. Care provided questionably not in the patient's best interests. A desire to treat patients beneficently.	Bordignon, Lunardi, Barlem et al (2018). Escolar, Magpantay (2019). Gandossi et al. (2023). Sasso, Bagnasco, Bianchi et al (2016). Wojtowicz, Hagen, Van Daalen‐Smith (2014). Yilmaz and Kiziltepe (2022).
Moral Courage.	Being able to perform virtuous actions. Demonstrating patient advocacy. Carrying out administrative actions of courage (e.g incident reporting). Morally courageous actions being ignored. Not being able to speak up and be morally courageous. Passive spectators of immoral actions.	Bickhoff, Sinclair, Levett‐ Jones (2017) Feeg, Mancino, Rushton et al. (2021) Krautsheid, DeMeester, Orton et al. (2017) Mæland, Tingvatn, Rykkje et al. (2021)
Internal Constraints	Disempowerment students experience. Feeling unable to challenge dominant characters in the hierarchy. Feeling they lack courage. Lack of confidence (nursing knowledge, nursing skills). Feeling they lack experience. Fear of repercussions. Fearing any backlash for their learning opportunities. Feeling they are incompetent to challenge. Feeling they don't have the skills to challenge. Fearing conflict. Wanting to fit in as part of the clinical team. Influenced by relationship with supervisor. Seeking reassurance. Not knowing where to turn to. Fear of being judged, not seeking support. Being ‘Just a student’ feeling its not within their role to challenge the hierarchy. Avoiding ‘rocking the boat’. Desire to maintain learning opportunities. Desire to maintain good relationship with supervisor.	Bickhoff, Sinclair, Levett‐ Jones (2017) Bordignon, Lunardi, Barlem et al. (2018) Escolar – Chua (2018) Escolar Chua, Magpantay (2019) Feeg, Mancino, Rushton et al. (2021) Gandossi et al. (2023). Krautsheid, DeMeester, Orton et al (2017). Krautsheid, Mood, McLennon et al. (2020) Reader (2015). Renno, Ramos, Brito (2018) Wojtowicz, Hagen, Van Daalen‐Smith (2014) Yilmaz and Kiziltepe (2022).
Consequences of moral distress		
Negative feelings.	Negative feelings to oneself. Self‐blame. Depression. Suicide risk. Stress. Anxiety. Fear. Induced physical symptoms. Regret. Anger. Helplessness.	Bickhoff, Sinclair, Levett‐ Jones (2017) Escolar Chua, Magpantay (2019) Feeg, Mancino, Rushton et al. (2021) Mæland, Tingvatn, Rykkje et al. (2021) Paidipati, Lozano, West et al. (2023) Reader (2015). Sasso, Bagnasco, Bianchi et al. (2016)
Coping mechanisms.	Detachment and avoidance of morally distressing situations. Distracting the patient so to not talk about their condition. Quitting Nursing. Remaining silent. Using strategies to resist power.	Bickhoff, Sinclair, Levett‐ Jones (2017) Escolar – Chua (2018) Gandossi, De Brasi, Rosa et al. (2023) Gibson, Duke, Alfred (2020) Sasso, Bagnasco, Bianchi et al. (2016) Yilmaz and Kiziltepe (2022).
Positive effects.	Reflection on actions. Supporting each other. Gaining confidence by sharing experiences. Learning from moral distress. Moral resilience.	Bordignon, Lunardi, Barlem et al. (2018) Gandossi, De Brasi, Rosa et al. (2023) Gibson, Duke, Alfred (2020) Renno, Ramos, Brito (2018) Sasso, Bagnasco, Bianchi et al. (2016) Yilmaz and Kiziltepe (2022).

## 5 OVERVIEW OF THE CONCEPT

In the context of health care and nursing, Jameton's (1984) definition of moral distress, based upon his encounters with student nurses, is widely used. Jameton (1984) defines moral distress as negative feelings that arise when one knows the morally correct response to a situation but cannot act because of institutional or hierarchal constraints. The definition identifies not only the requirement of moral behaviour but recognizes constraints exist as barriers to performing moral behaviour which causes moral distress (Epstein et al., 2016; Fourie, 2016; Morley et al., 2019). Epstein and Hamric (2009) focused on the nature of the constraint, and ask if it is only external, or could be as a result of internal or personal constraints, causing MD through self‐doubt, lack of assertiveness, or lack of understanding. The literature refers to this as internal constraints.

Many other definitions, however, maintain the core elements as originally formed by Jameton (1984): being compelled to act in a way that one believes is morally wrong but feels powerless to change (Epstein et al., 2016). This concept analysis identified Jameton's (1984, 2013, 2017) definitions of moral distress were referred to most frequently in the student nurse literature reviewed (see Table 4). Other definitions used predominantly in the student nurse population have derived from work by Corley, 1995, 2001; McCarthy and Gastmans's (2015); Fourie (2016).

**TABLE 4:** Sources of definitions of Moral Distress used by papers reviewed in concept analysis.

Definition	Papers in concept analysis
Jameton A (1984) Nursing practice: the ethical issues. Englewood, NJ: Prentice‐Hall.	Bordignon, Lunardi, Barlem et al. (2019) Feeg, Mancino, Rushton et al. (2021) Gandossi, De Brasi, Rosa et al. (2023) Gibson, Duke, Alfred (2020) Krautsheid, Mood, McLennon et al. (2020) Mæland, Tingvatn, Rykkje et al. (2021) Mazzotta, DeMaria, Bove et al. (2022) Sasso, Bagnasco, Bianchi et al. (2016)
Corley M (1995) Moral distress of critical care nurses. Am. J. Crit. Care Vol 4, 280–285.	Gandossi, De Brasi, Rosa D et al. (2023)
Corley M, Elswick R, Gorman M, et al (2001) Development and evaluation of moral distress scale. J Adv Nurs 33(2): 250–256.	Renno, Ramos, Brito (2018)
Corley M (2002) Nurse moral distress: a proposed theory and research agenda. Nurs Ethics; 9: 636–650.	Escolar Chua R, Magpantay J (2019)
Corley M, Inick P, Elswick R, Jacbos M (2005) Nurse moral distress and ethical work environment. Nursing Ethics, 12(4), 381—390.	Bickhoff, Sinclair, Levett‐ Jones (2017)
Hanna D (2005). The lived experience of moral distress: Nurses who assisted with elective abortions. Research and Theory for Nursing Practice, 19(1), 95–124.	Feeg, Mancino, Rushton et al. (2021)
Canadian Nurses Association. (2008). Code of ethics for registered nurses. Ottawa, ON: Author.	Wojtowicz, Hagen, Van Daalen‐Smith (2014)
McCarthy J, Deady R, (2008) Moral distress reconsidered. Nurs Ethics, 15: 254–262.	Defilippis, Prati, Scascighini (2023)
Epstein E, Delgado S (2010) Understanding and Addressing Moral Distress. Online J. Issue Nurs. 15, 1.	Gandossi, De Brasi, Rosa et al. (2023) Krautsheid, DeMeester, Orton et al. (2017)
Hamric A, Borchers C, Epstein E (2012). Development and testing of an instrument to measure moral distress in healthcare professionals. AJOB Primary Research, 3(2): 1–9.	Defilippis, Prati, Scascighini (2023) Feeg, Mancino, Rushton et al (2021).
Huffman M, Rittenmeyer L (2012) ‘How professional nurses working in hospital environments experience moral distress: a systematic review,’ Critical Care Nursing Clinics of North America, 24 (1): 91–100.	Janatolmakan, Dabiry, Rezaeian et al. (2021)
Jameton A (2013) A reflection on moral distress in nursing together with a current application of the concept. J Bioeth Inq 10(3): 297–308.	Renno, Ramos, Brito (2018)
Hamric, A. (2014). A case study of moral distress. Journal of Hospice and Palliative Nursing, 16(8): 457–463.	Krautsheid, DeMeester, Orton et al. (2017)
McCarthy J, Gastmans C (2015). Moral distress: a review of the argument‐based nursing ethics literature. Nurs Ethics; 22(1): 131–152.	Bordignon, Lunardi, Barlem et al. (2019) Krautsheid, DeMeester, Orton et al. (2017) Mæland, Tingvatn, Rykkje et al. (2021) Mazzotta, DeMaria, Bove et al. (2022)
Fourie C, (2015) Moral distress and moral conflict in clinical ethics. Bioethics; 29: 91.	Defilippis, Prati, Scascighini (2023) Feeg, Mancino, Rushton et al. (2021) Janatolmakan, Dabiry, Rezaeian et al. (2021) Krautsheid, DeMeester, Orton et al. (2017)
Jameton A (2017) What moral distress in nursing history could suggest about the future of healthcare. AMA Journal of Ethics,19(6): 617–628	Paidipati, Lozano, West et al. (2023)
Renno M, Ramos F, Brito M, (2018) ‘Moral distress of nursing undergraduates: myth or reality?’ Nursing Ethics, 25 (3): 304–312.	Janatolmakan, Dabiry, Rezaeian et al. (2021)
Morley G, Ives J, Bradbury‐Jones C, et al (2019). What is ‘moral distress’? A narrative synthesis of the literature. Nurs Ethics; 26: 646.	Defilippis, Prati, Scascighini (2023)
Morley G, Bradbury‐Jones C, Ives J. (2020) What is ‘moral distress’ in nursing? A feminist empirical bioethics study. Nurs Ethics; 27(5): 1297–1314.	Kane, Wareing, Rintakorpe (2022)
Webster G, Baylis F (2000) Moral residue. In: Rubin SB and Zoloth L (eds) Margin of error: the ethics of mistakes in the practice of medicine. Hagerstown, MD: University Publishing Group, pp. 217–230.	Escolar – Chua R (2018)
Moral Distress not defined by referencing.	Bahramirad, Heshmatifar, Rad (2020) Bourdignon, Lunardi, Barlem et al. (2018) Mehdipour R, Mahlagha D, Monirosadat N (2019)

### 5.1 Antecedents of moral distress

Antecedents are not the same as the concept itself but are a prerequisite for the concept to be experienced (Walker & Avant, 2005). Antecedents were revealed as students having moral sensitivity, the ability to recognize a moral conflict, show a contextual and intuitive understanding of the patient's vulnerable situation, and have insight into the ethical consequences of the decision on behalf of the person (Lutzen et al., 1995). The literature describes this as students having a cognitive rejection of nursing conditions which are internalized and viewed as morally reprehensible (Bongiorno et al., 2023). It involves students witnessing disheartening situations (Escolar Chua & Magpantay, 2019). Students knew the experiences they witnessed compromised patient care (Bickhoff et al., 2017), they were dangerous practices (Bickhoff et al., 2017) and were actions or behaviour's that compromised the health and wellbeing of patients and themselves (Bongiorno et al., 2023).

Participating in professional ethics workshops can increase one's moral sensitivity as this enables a better understanding of moral issues (Bakyara et al., 2015). Students who demonstrated more ethics knowledge and had participated in a professional ethics workshop were found to have a higher mean Moral Distress score 2.53 times higher in students who participated in the workshop than those who did not (Janatolmakan et al., 2021).

### 5.2 Defining attributes of moral distress

Defining attributes demonstrate the cluster of characteristics that are most frequently associated with the concept undergoing analysis (Walker & Avant, 2005). Defining attributes emerged as the roots of moral distress, moral courage and internal constraints.

### 5.3 Roots of moral distress

Poor care featured as one of the most frequently encountered sources of moral distress in a sample of 293 students in a study by Escolar‐Chua (2018) and in a sample of 86 students in a study by Janatolmakan et al. (2021) using the Moral Distress Scale Revised (MDS‐R), a Likert scale instrument (Hamric et al., 2012). Mental health students witnessed an over emphasis to provide medication rather than talking to the patient about their mental health and concerns in clinical practice (Wojtowicz et al., 2014). On night shifts, students witnessed nurses deal with patients pain very late and this gave rise to moral distress (Bahramirad et al., 2020). Students felt the nursing and medical care was slacking on night duty and decisions were in appropriate and having ethical implications (Bahramirad et al., 2020). Students witnessed patients not being offered what they perceived as the best treatment and care, and they found it difficult to speak up about their concerns (Mæland et al., 2021). Disrespect for service users' rights, privacy and being subject to prejudice attained higher mean moral distress scores in a sample of 282 delayed nursing students (Mazzotta et al., 2022).

Poor communication also emerged as a subtheme that impacted on poor patient care. In a study of 499 nursing students, Bordignon et al. (2019) found poor communication impacting on patient care scored the second highest mean moral distress score generated in the group. In a mixed methods study by Defilippis et al. (2023) using the MDS‐R in 66 nursing students who worked during the COVID 19 pandemic, highest levels of moral distress were found to be due to a decrease in the quality of care due to poor communication between colleagues. Using hermeneutic phenomenology, Gandossi et al. (2023) found that nursing students also experience moral distress when a patient, family member or both, were unaware of the terminal clinical condition of the patient due to the lack of clear communication by physicians.

Qualitative studies elaborate and suggest students expressed feeling pressured to conform to the poor practices evident in the clinical environment, even if these practices could negatively impact the quality of patient care (Bickhoff et al., 2017). A poor‐quality mentor—student relationships was found to stimulate students in complying with unethical or dangerous practices (Bickhoff et al., 2017). The knowledge acquired in university to correctly perform nursing interventions was also put aside when the undergraduate was faced with precarious work conditions, such as inadequate physical space and lack of material resources, among others (Renno et al., 2018).

Being involved in the delivery of unsafe care has continued to be a source of moral distress in the student nurse population for over a decade.. Using the MDS‐R one of the most distressing scenarios for students was asking to provide care for a patient that they were not competent to do (Escolar‐Chua, 2018). These findings continue to emerge in more recent studies using both interpretivist and positivist approaches. Yilmaz, Kiziltepe (2022) found in a sample of 126 students using phenomenology that if students were assigned tasks beyond the scope of their responsibilities and authority this generated moral distress. Students suffered moral distress when they witnessed their own errors and sometimes when they witnessed the errors of healthcare professionals when neglecting their duties (Yilmaz, Kiziltepe 2022).The incompetence of others was also a source of moral distress for students. Defilippis et al. (2023) found one of the top three causes of moral distress was when students worked with a doctor or nurse who they believed not to be competent in the COVID 19 pandemic.

Poor working conditions were also the basis for experiencing moral distress (Bongiorno et al., 2023; Escolar‐Chua, 2018; Kane, Wareing, Rintakorpe 2022). Students working during the first wave of the COVID 19 pandemic who experienced a lack of PPE encountered safety concerns leading to moral distress (Bongiorno et al., 2023). Similar findings are given in a paper by Kane et al. (2022) who found this was contributed to by lack of clear guidance on PPE, perceptions of constant change, and inadequate policies. Students felt their health was not as valued and voiced they were not disposable or martyrs (Bongiorno et al., 2023).

Internal and external constraints, however, were not always distinct and the intertwined roots (Heng and Shorey 2023) of both constraints appear influenced by the disempowerment students experience by being perceived as lacking power. The hierarchy of clinical practice emerged as a source of moral distress for students. Bickhoff et al. (2017) identified students feeling subordinate, invisible and without credibility. This finding occurred in other studies. Reader (2015) found students used words and phrases such as ‘just a student’, ‘belittle’, ‘pushed back’, ‘disregarded’, ‘inadequate’, ‘weak link’, and ‘hindered’ to articulate their experience, particularly in the clinical setting (Reader, 2015).

Hierarchical relationships between doctors and nurses also caused students to experience moral distress (Sasso et al., 2016). In two papers, students felt inferior not only to doctors, but other healthcare professionals, and felt they had no power (Escolar Chua & Magpantay, 2019; Mæland et al., 2021). Students described being unable to challenge the hierarchical power structures surrounding physicians, when describing their experiences with the unit psychiatrists in a paper by Wojtowicz et al. (2014). Doctors' dominance inhibited students and others to challenge their immoral behaviours (Renno et al., 2018). Narratives in a study by Renno et al. (2018) capture the superiority that influenced Doctors practice:

‘I saw it very clearly; I saw doctors harming patients, doing things wrong, but no one says a thing because no one will contradict the doctor. I felt very bad because of this’ (Renno et al., 2018).

Moral distress also transpired when students witnessed inconsistency between theory against practice (Bahramirad et al., 2020). However, extracts from a student in a study by Renno et al. (2018) reveal the subordinance they experienced being bottom of the hierarchy:

‘They apply the dressing in a way, they use certain dressings, and as undergraduates we know it's not the ideal ones, we know it's wrong, but we can't say anything’ (Renno et al., 2018).

The power and hierarchy experienced by students in the academic environment was also a source of moral distress. Lack of teachers' ethical attitude, (Renno et al., 2018), incivility (Sasso et al., 2016) and authoritarian teaching practices generated moral distress (Bordignon et al., 2019). Using the Moral Distress Scale adapted for students, studies found that students who had previously failed an assessment had higher levels of moral distress in the construct of authoritarian teaching practices (Bordignon et al., 2019; Mazzotta, DeMaria, Bove et al 2022). Negative behaviours of academic staff included threatening, punishing, stigmatizing some students by professors which caused moral distress (Janatolmakan et al., 2021). Humiliation of students by academic staff was the foundation for moral distress in papers by Renno et al. (2018); Janatolmakan et al. (2021). Students referred to learning in an unjust culture and used narratives such as unfair, fear, target, retaliation, blacklisted, black mark, backlash, harassment, intimidation, and blame (Reader, 2015). In a paper by Reader (2015) a student referred to some professors watching students, suggesting teachers were out to get students quoting:

‘I do feel like, not only in this institution, but other institutions, there are teachers out there who are looking for blood.’

Academics lack of teaching pedagogy and a focus instead on research was also found to cause moral distress for student nurses in the academic environment (Renno et al., 2018). Improper institutional conditions to teach patient care was a high source of moral distress for students (Bordignon et al., 2019).

### 5.4 Moral courage

When witness to morally reprehensible situations, students believed they knew the righteous thing to do. On some occasions they performed virtuous actions which enabled them to do the right thing (Bordignon et al., 2018; Feeg et al., 2021). On other occasions students should have acted but did not, this was due to cognitive barriers such as lack of confidence (Krautsheid et al., 2017), courage (Bickhoff et al., 2017), and fear of the repercussions of conflict (Bordignon et al., 2018). Following analysis of 36 stories of challenging clinical practice experiences, Mæland et al. (2021) concluded students knew the right action in the situation but ended up doing nothing (Mæland et al., 2021).

Moral courage is the willingness to take a stand that challenges the health care organization or those in it, even when a person's job may be jeopardized (Fry, 1989). Despite feeling a moral obligation to act, some papers suggest some nursing students lacked the moral courage to speak up when it was required the most, causing moral distress (Bickhoff et al., 2017). Whilst students may report the negative behaviour, after the event, they often remain passive spectators and sometimes even active participants at the time (Bickhoff et al., 2017). Feeg et al. (2021) recognizes some of the actions students took to report immoral incidents included incident reporting, filing a grievance, patient advocacy and ‘sticking up for patients’ against medical staff (Feeg et al., 2021). However, despite students attempts to speak up and advocate for quality patient care, narratives reveal how the students were ignored or belittled (Krautsheid et al., 2017). Students who demonstrated more moral courage experienced moral distress less frequently, however, when this was experienced it was encountered more extremely (Jantara et al., 2023).

### 5.5 Internal constraints

Internal constraints are constraints located within the individuals themselves and are recognized as personal limitations, failings or weakness of will (Deschnes et al., 2020). Students inaction was found to be as a result of cognitive internal constraints such as the student's desire to avoid conflict and avoid ‘rocking the boat’ (Bickhoff et al., 2017; Escolar Chua & Magpantay, 2019), to preserve the field of practical activity and preserve opportunities for learning (Bickhoff et al., 2017; Bordignon et al., 2018), preserve relationships with their supervisors (Krautsheid et al., 2017; Krautsheid et al., 2020).

Lack of confidence emerged as a theme contributing to inaction and was evident in several studies (Bickhoff et al., 2017; Bordignon et al., 2018; Escolar‐Chua, 2018; Krautsheid et al., 2020; Yilmaz, Kiziltepe 2022). Nursing students were hesitant to voice their concerns despite their role of being patient advocates. Students' failure to act was due to their lack of confidence and competence to question the unethical behaviour of others as well as avoiding conflict within the team (Bickhoff et al., 2017; Escolar Chua & Magpantay, 2019). Confidence was also linked to knowledge. Lack of knowledge manifested in not knowing how to respectfully speak up to the person(s) involved with the immoral event (Krautsheid et al., 2017).A desire to maintain a good relationship with professors, and a lack of self‐confidence to own knowledge were the main reasons for moral distress in a study by Krautsheid et al. (2020). Students who felt more confident, however, reported issues in the clinical practice (Bickhoff et al., 2017), and academic environment (Bordignon et al., 2018). Students lack in confidence was evident in that they looked to faculty to validate their concerns were correct (Feeg et al., 2021).

The power differential between Registered Nurses (RNs) and students was the most common theme in the literature which effected whether a student would act (Bickhoff et al., 2017). Students desire to be accepted as part of the practice team was compelling to the point where students reported compromising their own morals and beliefs to fit in with their clinical practice area (Bickhoff et al., 2017). Positive relationships between RN's and students resulted in students who were more likely to challenge poor practices (Bickhoff et al., 2017), however, poor quality mentor—student relationships led students to complying with unethical or dangerous practices (Bickhoff et al., 2017). Students repeatedly diminished their role on clinical placements to that of being ‘just a student’, thereby creating a perception that it was not within their scope to question the practice of (RN's) (Bickhoff et al., 2017; Escolar Chua & Magpantay, 2019; Reader, 2015).

‘Just a student’ theme was also evident in the academic environment (Bordignon et al., 2018); Reader (2015). Bordignon et al. (2018) presented extracts from students who experienced moral distress because of their own inaction in University:

‘How would I tell the teacher that I did not agree? … professors are always right, and we can't say the same about students… What am I? Only a graduate student, nobody special’ (Bordignon et al., 2018).

Consequently, being ‘just a student’ compelled students to remain ‘silent in the world of just being a student’ and did not raise concerns or act upon their concerns (Bickhoff et al., 2017). Students reported that their supervising RNs also referred to them as ‘just’ or ‘only’ a student, thereby reinforcing this subordinate lack of credible identity (Bickhoff et al., 2017).

### 5.6 Consequences of MD

Consequences are those events that occur because of the concept occurring (Walker & Avant, 2005 p73). Five themes emerged in the analysis and included negative feelings to self and profession, distancing themselves emotionally as a means of coping, reflections enabling learning from moral distress, gaining support from each other and strategies of resistance.

Students reported negative feelings such as feeling responsible and blaming themselves for any negative outcomes or distress the patient suffered even if they were only witness to and not active participants in the situation (Bickhoff et al., 2017; Feeg et al., 2021; Reader, 2015). Witnessing situations that caused moral distress left students to have feelings of sadness and disappointment (Escolar Chua & Magpantay, 2019). Reader (2015) suggested every narrative in their study included elements of regret and the lingering effects of moral distress. Students used terms such as ‘carry for the rest of my life’, ‘I should have’, ‘I wish I had’, ‘I didn't speak up’,’ I didn't say anything’, ‘didn't know what to do’, ‘I am still bothered’ to describe their experiences, how they felt, and still feel. (Reader, 2015). This theme transpired in other papers: ‘I was so disappointed also in myself, that I was not able to do anything to correct them because I immediately thought that I was inferior to them. I had no power’ (Escolar Chua & Magpantay, 2019).Other studies referred to student narratives suggesting a lack of courage: ‘I felt like a coward. It's like I lost my voice’ (Escolar Chua & Magpantay, 2019).

Moral distress had a physical and emotional impact on many students. They reported symptoms such as not being able to sleep at night, feeling stressed and anxious at the thought of returning to the ward, and even vomiting (Bickhoff et al., 2017). Physical symptoms, such as sleep disorders, headache, agitation, gastrointestinal problems were suffered by students (Sasso et al., 2016). Psychological symptoms, such as feelings of anguish, frustration, anxiety, or guilt, which may even result in burnout and emotional breakdown were also encountered (Sasso et al., 2016). Students described going ‘home at the end of shift in tears’ and dreading going back every day (Bickhoff et al., 2017). A sense of loneliness was felt by students (Sasso et al., 2016). Moral distress was found to be significantly associated with both suicide risk and depression (Paidipati et al., 2023). Some students found their clinical training experience so discouraging that they were no longer willing to work in the field of mental health (Sasso et al., 2016). Some students desired to discontinue their studies (Sasso et al., 2016, Bickhoff et al., 2017; Yilmaz, Kiziltepe 2022). However, in other papers, despite frequently encountering moral distress 79.2% of the respondents hardly consider quitting the nursing profession (Escolar‐Chua, 2018). A similar finding was reported in a study by Gibson et al. (2020) who found although students had mild moral distress, 82.2% had not considered quitting nursing school.

Nursing students observed that some RNs seemed cold and a little standoffish even though they realized that their attitude was not due to a lack of sensitivity but to the attempt to avoid being involved emotionally with dying patients (Gandossi et al., 2023). Students also avoided caring for patients when they experienced moral distress (Yilmaz, Kiziltepe 2022), and distanced themselves, both physically and emotionally, as a coping mechanism in response to practices and treatment of patients that went against their moral and professional values (Bickhoff et al., 2017). Trainees often worried that they might not be able to maintain a compassionate attitude once they have become professional nurses (Sasso et al., 2016).

The experience of moral distress encouraged students to reflection on their encounters. Many nursing students' re‐thought reflected and re‐evaluated morally distressing events. They wondered if they had done their best and made the right choice (Gandossi et al., 2023). This process allowed them to re‐evaluate their actions and professionals' actions, wondering how they would have acted if they had been in their place (Gandossi et al., 2023).

When faced with situations requiring a compassionate approach to help them deal with their events, trainees sought support from those around them, whether that be other trainees, family members, mentors, or university tutors. (Sasso et al., 2016; Yilmaz, Kiziltepe 2022). Students reported that support is essential to control their feelings and cope with emotionally challenging events during their clinical training (Sasso et al., 2016). Nevertheless, it appears that this support is available only if the student explicitly asks for it (Sasso et al., 2016). Students moral distress was relieved when their point of view on a situation coincided with that of the care team, making them feel supported and safe (Gandossi et al., 2023). Many students realized they needed to talk to other people and reflect on their experience, wondering how they could have acted differently (Gandossi et al., 2023). Many nursing students highlighted that the consultation after a morally distressing event with an internship assistant was useful, they felt supported and understood and it helped them to better deal with their malaise (Gandossi et al., 2023). Some students referred to moral distress being part of the construction of their political education and thought moral distress was a necessary experience (Renno et al., 2018), and some had developed learning from MD (Gandossi et al., 2023). Some nursing students, however, finally preferred not to consult anyone due to fear of being judged and lacking moral courage (Gandossi et al., 2023).

Some students coped with morally inappropriate situations by being assertive and exhibited their reaction through acts of resistance. This however was influenced by their perceived confidence and knowledge. Students who experienced moral distress in the academic environment resisted academic power when they realized they had more mastery of nursing knowledge, resulting in greater security and confidence to carry out confrontations and proving their moral strengthening (Bordignon et al., 2018). Students' resistance to power strategies included getting together as a group and complain about the professor (Bordignon et al., 2018). Some colleagues sued the university, and the professor was fired (Bordignon et al., 2018). They participated in research to voice their experiences as a means of dealing with the situation and used rating and feedback opportunities of professors to express their voice (Bordignon et al., 2018).

### 5.7 Define empirical referents

Determining the empirical referents for the defining attributes of the concept in the final step in a concept analysis and helps us to identify if we are to measure this concept or determine its existence in the real world, how would we do it? (Walker & Avant, 2005). Defining empirical referents are useful in addressing content and construct validity in instrument development as they are clearly linked to the theoretical base of the concept (Walker & Avant, 2005).

The analysis has identified several empirical referents to measure moral distress in student nurses:

1. Student is witness to a morally reprehensible event.
2. Roots of moral distress include poor care, poor communication, being involved in unsafe care, poor working conditions, inconsistency between theory and practice, hierarchical structures, and incivility. This is exacerbated by their perceived disempowerment.
3. Student recognizes an event as morally reprehensible in either the academic or clinical practice environment and feels they have a responsibility to act upon this.
4. The student fails to act on the immoral event at the time due to a combination of cognitive barriers/ internal constraints and /or institutional constraints. Internal constraints include avoiding conflict, preserving learning, relationship with supervisor, lack of confidence, feeling subordinate, not feeling credible, perceived/ actual lack of competence and knowledge. Some actions to address the immoral behaviour may be taken after the event.
5. Student experiences negative feelings (sadness, disappointment, self‐blame, regret, feeling cowardly, guilt, negative stress, physical symptoms, psychological symptoms, anxiety, depression, suicide, loneliness, avoidance physically and emotionally, concern for future compassionate fatigue, leaving the profession).
6. The student may experience positive consequences of moral distress in addition to initial negative feelings (reflective learning, gaining support), engages with strategies of resistance to reduce moral distress (e.g. reporting professor).

### 5.8 Identify a model case

A model case is an example of the use of the concept that demonstrates all of the attributes of the concept (Walker & Avant, 2005), and uses an extract taken from Participant 3 in a paper by Escolar Chua and Magpantay (2019):

‘Not everyone is treated equally in terms of their financial standing. I was questioning a lot of things to my instructor because it's not how we were taught. That's not what I think we were taught’.

‘I wasn't able to say anything. My group mates as well, we didn't say anything. I felt like a coward. It's like I lost my voice.’

This participant would then experience negative feelings and may experience positive feelings thereafter.

### 5.9 Identify illegitimate cases

These are examples of terms used improperly or out of context (Walker & Avant, 2005).

If students are unable to identify that a morally reprehensible event occurs and/or does not suffer distress in response to being witness to a morally reprehensible event, then moral distress cannot occur.

Renno et al. (2018) recognized in their study that some experiences, practiced or observed, cause unpleasant feelings for students that, cannot be characterized as moral distress, but they were reported by the undergraduates, who recognize that their susceptibility might be exacerbated by inexperience. Renno et al. (2018) however accept they cannot define an experience of moral distress only when it is considered to be so by a nurse. Moral distress is therefore evident if the student perceives it to constitute moral distress.

### 5.10 Advantages/ limitations of the analysis

Advantages are that the analysis did not exclude specific epistemologies or methodologies by using an integrated mixed research synthesis (Sandelowski et al., 2006). The analysis included current peer reviewed papers, thereby enhancing the trustworthiness of the analysis. Current papers within 10 years of publication were included and represented current evidence base. Whilst a literature review was included in the analysis; primary research papers supported the literature review findings. Limitations give reference to the inclusion criteria not considering papers in English language which excluded three papers. A further limitation is that whilst concept analysis must be rigorous and precise; the product is always tentative as evidence base changes (McKenna, 2014).

### 5.11 Trustworthiness

The researcher utilized a variety of methods to establish trustworthiness as discussed by Lincoln and Guba (1985). These methods include triangulation by using multiple sources and methods of literature. The researcher practiced peer debriefing with her experienced doctoral supervisors during the analysis (Lincoln & Guba, 1985). The author also used reflexive journalling with the aim of averting researchers' biases influencing interpretations and outcomes.

## 6 DISCUSSION

Despite different frameworks being used for analysis, this concept analysis has identified some similarities and differences in how student nurses experience moral distress in comparison to concept analysis of Critical Care Nurses and Midwives. Whilst antecedents of moral distress differ across all papers, key similarities are that the Student Nurse, Nurse and Midwife possess moral sensitivity and can recognize ethical circumstances in their clinical practice.

There was, however, a notable difference in the attributes of moral distress within student nurses' experiences. Whilst critical care nurses moral distress stemmed from nurses' perceptions of futile care contributing to prolonging patient suffering (Cooke et al, 2022). Midwives' sources of moral distress, however, were often in the context of their moral uncertainty related to termination of pregnancy and removal of babies form their mothers which conflicted with their professional values (Foster, 2022). Distinguishing sources of moral distress in the student nurse population derived from being involved in unsafe care and inconsistency between theory and practice which is exacerbated by their experience of disempowerment.

Foster (2022) also recognized that Midwives did not always challenge care they felt was detrimental to the woman which impacted on their moral distress. Midwives also felt obliged to uphold the decisions made by medical staff, which was attributed by hierarchical structures, even though the midwife disagreed with these decisions (Foster, 2022). Attributes of moral distress in the student nurse population also identify internal constraints inhibiting moral courage but are due to a fear of conflict, and to preserve their learning opportunities and is influenced by their relationship with their nursing supervisor. Whether the student will take action when witness to immoral events is influenced by their lack of confidence, and credibility by being ‘just a student’ and perceived, or actual lack of competence and knowledge. These attributes are distinguishable to students in the context of their extraordinary position in clinical practice.

Concept analysis performed by Cooke (2022) in Critical Care Nurses, and Foster (2022) in Midwives also identified negative and positive impacts of moral distress similar to that experienced by student nurses. Negative emotional effects included feelings of anxiety, sadness, guilt (Cooke 2022), fear, and confusion (Foster, 2022). Positive consequences were found to also include reflection (Cooke 2022). Nurses and midwives, like students in this analysis also expressed an intention to leave their profession (Cooke 2022, Foster, 2022).

Progress has been made to adapt instrumentation used to investigate moral distress in the student nurse population. Further adaptions considering the analysis findings, and in particular the contextual and distinguishing attributes that influence whether a student will or will not act when witness to immoral events, could be incorporated to investigate internal constraints which can hinder students exhibiting moral courage.

## 7 CONCLUSION

This is the first concept analysis performed in the student nurse population which explores their experiences of moral distress. In the context of their role as a student nurse, students have distinguishing attributes of moral distress and these differences should be considered by nurse educatiors, and nurses supporting student nurses in clinical practice settings to support students in addressing moral distress. The findings of this analysis can also aid development of empirical research and addressing moral distress through evidence based educational interventions.

## AUTHOR CONTRIBUTIONS

TIMMINS Rebecca: Substantial contributions to the conception and design/methodology of the work. To investigation: literature searching, reviewing analysis, and interpretation of data for the work. To data curation. To drafting the work and reviewing it critically for important intellectual content; AND final approval of the version to be published; and Agreement to be accountable for all aspects of the work in ensuring that questions related to the accuracy or integrity of any part of the work are appropriately investigated and resolved.

## FUNDING INFORMATION

University of Wolverhampton (EDd funding course fees, no grant number).

## CONFLICT OF INTEREST STATEMENT

No conflicts of interests declared.

## ETHICS STATEMENT

None as this is not empirical research.

## Data Availability

The data that support the findings of this study are available from the corresponding author upon reasonable request.
